# Dietary Tryptophan Supplementation Improves Antioxidant Status and Alleviates Inflammation, Endoplasmic Reticulum Stress, Apoptosis, and Pyroptosis in the Intestine of Piglets after Lipopolysaccharide Challenge

**DOI:** 10.3390/antiox11050872

**Published:** 2022-04-28

**Authors:** Guangmang Liu, Jingyuan Tao, Jiajia Lu, Gang Jia, Hua Zhao, Xiaoling Chen, Gang Tian, Jingyi Cai, Ruinan Zhang, Jing Wang

**Affiliations:** 1 Institute of Animal Nutrition, Key Laboratory for Animal Disease-Resistance Nutrition, Ministry of Education, Ministry of Agriculture and Rural Affairs, Key Laboratory of Sichuan Province, Sichuan Agricultural University, Chengdu 611130, China; taojingyuan526@163.com (J.T.); ljj840931546@163.com (J.L.); jiagang700510@163.com (G.J.); zhua666@126.com (H.Z.); xlchen@sicau.edu.cn (X.C.); tgang2008@126.com (G.T.); jycai2004@aliyun.com (J.C.); 2 Maize Research Institute, Sichuan Agricultural University, Chengdu 611130, China; wangj221@gmail.com

**Keywords:** tryptophan, antioxidant status, endoplasmic reticulum stress, apoptosis, pyroptosis, pig

## Abstract

Tryptophan can alleviate stress and improve intestinal health, but the precise mechanism has not been fully elucidated. This study aimed to examine the effects of tryptophan supplementation on antioxidant status, inflammation, endoplasmic reticulum (ER) stress, apoptosis, and pyroptosis signaling pathway in the intestine of piglets after *Escherichia coli* lipopolysaccharide (LPS) challenge. Thirty-two weaning piglets were allotted to four treatments including: non-challenged control, LPS-challenged control, LPS + 0.2% tryptophan and LPS + 0.4% tryptophan. On day 35 of feeding, piglets were injected intraperitoneally with 100 μg/kg of body weight LPS or saline. Among the LPS-challenged pigs, tryptophan supplementation improved intestinal morphology as indicated by greater villus height, villus area and smaller crypt depth, and antioxidant status, and decreased the mRNA expression and concentration of proinflammatory cytokines. Moreover, tryptophan downregulated the expression of ER stress (ER oxidoreductase-1α, ER oxidoreductase-1β, glucose-regulated protein-78, activating transcription factor 6, C/EBP homologous protein), apoptosis (B-cell lymphoma-2, BCL2-associated X protein, caspase 3), and pyroptosis signaling pathway (nucleotide-binding oligomerization domain-like receptor protein 3, caspase 1, gasdermin-D, apoptosis-associated speck-like protein containing a CARD). Collectively, tryptophan supplementation can contribute to gut health by improving antioxidant status and alleviating inflammation, ER stress, apoptosis, and pyroptosis in the intestine of piglets after lipopolysaccharide challenge.

## 1. Introduction

The intestine epithelium is not only critical for the digestion and absorption of nutrients but is also an effective barrier against bacteria-derived endogenous and exogenous harmful agents [1]. The overproduction of reactive oxygen species (ROS) can increase the expression of proinflammatory cytokines and induce endoplasmic reticulum (ER) stress, apoptosis, and pyroptosis [2,3]. Apoptosis and pyroptosis are important models of programmed cell death. The activation of inflammasomes and gasdermin-D (GSDMD) can lead to pyroptosis. Pyroptosis is proinflammatory, causing the spread of inflammation. Excessive inflammation, ER stress, apoptosis, and pyroptosis can cause intestinal dysfunction [4,5]. The dysfunction of the intestinal mucosal barrier aggravates gut permeability, inflammatory response, ER stress, apoptosis, and pyroptosis, leading to bacterial translocation and subsequent intestinal infection [6]. Nutritional interventions may exert beneficial effects in abrogating ER stress to restore ER function, attenuating epithelial apoptosis and pyroptosis, reducing intestinal mucosal inflammation, and preserving intestinal function [6,7].

Tryptophan is usually considered the second or third limiting amino acid in pig diets. Tryptophan can enhance the growth, mitochondrial function, and antioxidant capacity, improve immunity, increase the diversity of the intestinal microbiome and cell migration, and protect the intestinal integrity in animals [7,8,9,10,11,12,13,14]. Insufficient tryptophan supplementation will reduce the absorption and use of protein and the immune function of livestock and poultry and increase the susceptibility of livestock and poultry to diseases. Inflammation can induce tryptophan metabolism, and tryptophan can be mainly catabolized to the kynurenine pathway by indoleamine 2,3 dioxygenase (IDO). 5-Hydroxytryptamine (5-HT) and melatonin (MT) are important metabolites of tryptophan. 5-HT can enhance host immune function by inhibiting the production of peroxides and tumor necrosis factor-α (TNF-α) and scavenging free radicals [15]. MT has been shown to have antioxidant activities both in vivo and in vitro, acting as a scavenger of ROS [16]. Because of antioxidant characteristics, MT influences cell viability by modulating the ER stress response [17]. However, the effects of tryptophan supplementation on intestinal ER stress have not been investigated. No information is available about tryptophan supplementation on pyroptosis in mammalian species. This study aimed to confirm whether tryptophan supplementation can improve antioxidant status and alleviates inflammation, ER stress, apoptosis, and pyroptosis in the intestine of piglets after lipopolysaccharide challenge. The experimental results can provide a theoretical basis for the rational application of tryptophan in weaned piglet diets and also provide new strategies to regulate the intestinal immune function of weaned piglets by means of nutrition.

## 2. Materials and Methods

### 2.1. Pig Care and Experimental Design

Feeding management in this study was conducted in accordance with the guidelines established by Sichuan Agricultural University Animal Care and Use Committee. A total of 24 castrated barrows (Duroc × Large White × Landrace; weaned at 24 ± 1 days of age) were randomly divided into four treatments with eight replicates per group. During the whole experiment, the controlled room temperature was maintained at approximately 30 °C, and the relative humidity ranged from 50% to 60%. Every piglet was supplied with clean drinking water. The basal diet (Table 1) was formulated according to National Research Council (2012) requirements for all nutrients.

The experiment included four treatments as follows: (1) non-challenged control (CONTR; the pigs were fed a basal diet and injected with 0.9% saline); (2) lipopolysaccharide (LPS)-challenged control (the pigs were fed with the same control diet and injected with *E. coli* LPS); (3) LPS + 0.2% Trp treatment (the pigs were fed with a 0.2% l-Tryptophan (Trp, purity > 99%; CJ International Trading Co., Ltd. Seoul, Korea) diet and injected with *E. coli* LPS); and (4) LPS + 0.4% Trp treatment (the pigs were fed with a 0.4% Trp diet and injected with *E. coli* LPS). On day 35 of the trial, the LPS, LPS + 0.2% Trp, and LPS + 0.4% Trp groups were injected intraperitoneally with 100 μg/kg BW *E. coli* LPS (*E. coli* serotype 055: B5; Sigma Chemical Inc., St. Louis, MO, USA), and the CONTR group was injected with the same volume of 0.9% (*w*/*v*) saline; the dose of LPS was chosen as described by Zhu et al. [18]. To avoid feed intake change, which has the potential influence on the intestinal mucosa, we deprived all piglets of feed for 4 h until slaughter.

### 2.2. Intestinal Sample Collections

On the 35th day of the trial, we chose the time point of 4 h after injection with saline or LPS injection for sample collection. The ileal tissues (approximately 5 cm) were washed in 0.9% saline and fixed in 4% paraformaldehydethen. Then, the samples were rapidly frozen in liquid nitrogen and stored at −80 °C for histologic analysis.

### 2.3. Intestinal Morphology Analysis

After 24 h of fixation, fixed intestinal samples were prepared based on the conventional paraffin-embedding techniques. In brief, cross-sections of the segments were cut into 4 μm-thick sections by using a microtome (Leica Instrument Co., Ltd., Shanghai, China) and stained with hematoxylin and eosin (H&E) (Servicebio Technology Co., Ltd., Wuhan, China). The villus height and related crypt depth were measured. The method for confirmability of villus height and crypt depth is based on a previous study [19].

### 2.4. Measurement of the Antioxidant Parameters

The antioxidant activities were determined by employing commercially available kits (Jiancheng Bioengineering Institute, Nanjing, China). In brief, malondialdehyde (MDA) content was estimated using the thiobarbituric method (TBA method) as described by Cao et al. [20]. Catalase (CAT) was evaluated using a colorimetric method [19]. The total superoxide dismutase (T-SOD) activity was analyzed as described by Cao et al. [20].

### 2.5. Real-time PCR Analysis

The method of the detection and analysis of RNA from the ileal tissue was based on a previous study [17]. Briefly, total RNA in the ileum was used to synthesize cDNA by using the Prime Script™ RT reagent (Takara, Dalian, China). RNA was determined using a fluorometric assay with the SYBR^®^ Primix Ex Taq II kit (Takara, Dalian, China). The specific primers of the genes involved in our experiment were designed on the Primer Express Software (version 3.0; Applied Biosystems, Foster City, CA, USA) and composited by Takara Biotechnology Company (Takara, Dalian, China). Table 2 lists the forward and reverse primers of the correlative genes.

### 2.6. Western Blot Assay

The steps are based on the procedures used by Liu et al. [21]. In brief, in keeping with the manufacturer’s protocol, total protein concentration was detected using the BCA kit obtained from the Beyotime Institute of Biotechnology (Shanghai, China). Samples in the ileal mucosa were obtained via SDS-PAGE and transferred to PVDF membranes (Millipore, Eschborn, Germany). The membrane was blocked and then incubated with primary antibodies overnight (4 °C). The membrane was rinsed and then incubated with the secondary antibodies for 60 min (25 °C). The antibodies, including glucose-regulated protein-78 (GRP78), activating transcription factor 6 (ATF6), gasdermin-D (GSDMD), caspase-1, caspase 3, and nucleotide-binding oligomerization domain-like receptor protein 3 (NLRP3) conjugated anti-rabbit antibodies were supplied by Proteintech Group, Inc. (Wuhan, China). The signal was visualized by extreme hypersensitivity ECL chemiluminescence kit (Beyotime, Shanghai, China), and the value was displayed using the Image lab analysis software (version 6.1, Bio-Rad. Berkeley, CA, USA).

### 2.7. Statistical Analysis

Data were analyzed using SPSS 26.0 (SPSS Inc., Chicago, IL, USA). Values of *p* < 0.05 were used to denote statistically significant differences between the groups. Planned contrasts were used for comparison as follows: (1) LPS pigs were compared with CONTR pigs to determine the effect of LPS challenge pigs; (2) the different dose-response effects of tryptophan were tested employing linear and quadratic trends for the three tryptophan levels (0%, 0.2%, and 0.4% tryptophan) among piglets injected with LPS. Results are expressed as mean and pooled SEM.

## 3. Results

### 3.1. Intestinal Morphology

**Figure 1:**
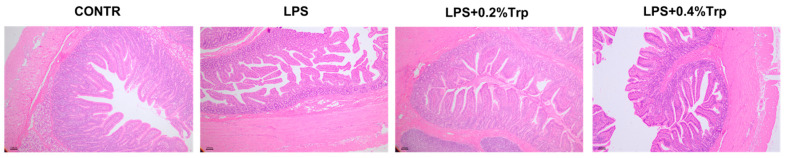
Effect of tryptophan supplementation on ileal morphology after 4 h LPS challenge in piglets. CONTR, control; LPS, lipopolysaccharide; Trp, tryptophan. CONTR (non-challenged control), piglets were supplemented with a basal diet and injected with 0.9% NaCl solution; LPS (LPS-challenged control), piglets were supplemented with the same basal diet and injected with *E. coli* LPS; LPS + 0.2% Trp, piglets fed a 0.2% tryptophan-supplemented diet as well as injected with LPS; LPS + 0.4% Trp, piglets fed a 0.4% tryptophan-supplemented diet as well as injected with LPS.

In comparison with CONTR pigs, LPS challenge decreased ileal villus height (*p* < 0.001), villus area (*p* < 0.05) and ileal villus height/crypt depth ratio (VCR) (*p* < 0.05; Table 3; Figure 1). Among the LPS-challenged pigs, Trp supplementation increased ileal villus height (linear, *p* < 0.05), VCR (linear, *p* < 0.01), and villus area (linear, *p* < 0.05), and decreased crypt depth (linear, *p* < 0.05).

### 3.2. Antioxidant Indicators in the Ileum

Table 4 shows the antioxidant indicators in the ileum. In comparison with CONTR pigs, LPS challenge increased the ileal malondialdehyde (MDA), hydrogen peroxide (H_2_O_2_) and reactive oxygen species (ROS) content (*p* < 0.05) and decreased the ileal total superoxide dismutase (T-SOD), catalase (CAT), glutathione peroxidase (GSH-Px) activity (*p* < 0.05). Among the LPS-challenged pigs, Trp supplementation remarkably decreased ileal MDA content (quadratic, *p* < 0.05), H_2_O_2_ concentration (linear, *p* < 0.001; quadratic, *p* < 0.001) and ROS content (linear, *p* < 0.001; quadratic, *p* < 0.001), and increased ileal CAT content (linear, *p* < 0.001; quadratic, *p* < 0.05), GSH-Px (linear, *p* < 0.001; quadratic, *p* < 0.001), and T-SOD activity (linear, *p* < 0.05; quadratic, *p* < 0.05).

### 3.3. The Concentration and mRNA Expression of Ileal Cytokines and Activity of IDO

The data for the activity of IDO and cytokine concentration of TNF-α, interleukin (IL)-1β, IL-6, IL-8, IL-10, and interferon-γ (IFN-γ) are shown in Table 5. Relative to CONTR pigs, the LPS pigs had higher activity of IDO and concentrations of TNF-α, IL-1β, IL-6, IL-8, and IFN-γ in the ileum (*p* < 0.001). In the LPS-challenged pigs, both the dietary supplementation of the 0.2% Trp and the 0.4% Trp pigs had lower activity of IDO (linear, *p* < 0.01; quadratic, *p* < 0.001) and concentrations of TNF-α, IL-1β, IL-6, IL-8 (linear, *p* < 0.001; quadratic, *p* < 0.001) and IFN-γ (linear, *p* < 0.05; quadratic, *p* < 0.001) in ileum. Compared with CONTR pigs, the LPS pigs’ concentration of IL-10 significantly decreased (*p* < 0.001). Among the LPS-challenged pigs, Trp supplementation increased the concentration of IL-10 in the ileum (linear, *p* < 0.001). The data for the mRNA expression levels of IL-1β, IL-6, IL-8, IL-18, and TNF-α are shown in Table 6. The LPS pigs had higher mRNA expression levels of IL-1β, IL-6, IL-8, IL-18, and TNF-α in the ileum relative to CONTR pigs (*p* < 0.05). In the LPS-challenged pigs, both the dietary supplementation of 0.2% Trp and 0.4% Trp pigs decreased IL-1β, IL-6, IL-8, IL-18, and TNF-α mRNA abundance in the ileum (linear, *p* < 0.01; quadratic, *p* < 0.05).

### 3.4. mRNA Expression Levels of ER Stress-, Pyroptosis, and Apoptosis-Related Genes in Ileal Tissues

The mRNA expression levels of ER stress-, pyroptosis-, and apoptosis-related genes in ileal tissues are shown in Table 7. QRT-PCR results show that compared with CONTR pigs, the LPS pigs’ mRNA expression levels of endoplasmic reticulum oxidoreductase-1α (ERO1*α*, *p* < 0.05), caspase 1, GRP78, ATF6, endoplasmic reticulum oxidoreductase-1β (ERO1*β*), NLRP3, GSDMD, apoptosis-associated speck-like protein containing a CARD (ASC), B-cell lymphoma-2 (BCL-2), BCL2-associated X protein (Bax), caspase 3, and C/EBP homologous protein (CHOP) significantly increased (*p* < 0.05). Among the LPS-challenged pigs, Trp supplementation decreased the mRNA expression levels of ileal ERO1*α* (linear, *p* < 0.01), caspase 1, GRP78, ATF6, ERO1*β*, NLRP3, ASC, GSDMD, Bax, BCL-2, Caspase 3 and CHOP in the ileum (linear, *p* < 0.01; quadratic, *p* < 0.05).

### 3.5. ER Stress-, Pyroptosis-, and Apoptosis-Related Protein Expression Levels in Ileal Tissues of Piglets

**Figure 2:**
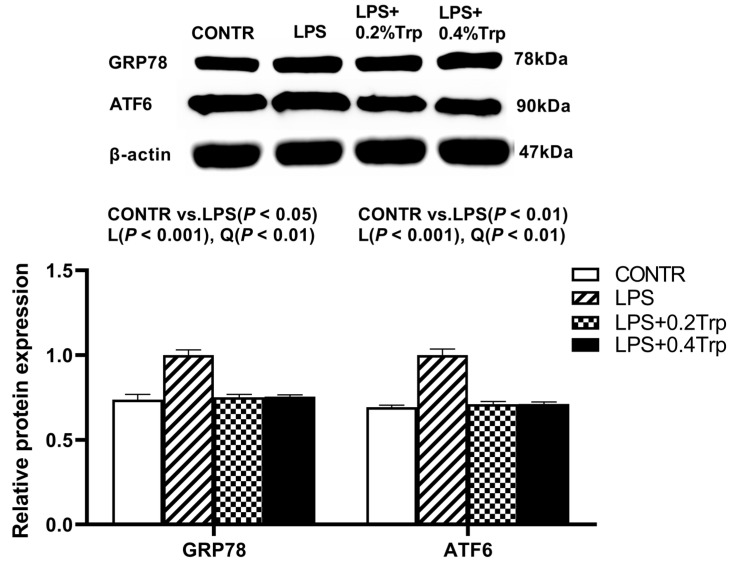
Impact of tryptophan supplementation on the ratios of GRP78/β-actin and ATF6/β-actin after 4 h of *E. coli* LPS challenge in ileal mucosa of weaning piglets. GRP78, glucose-regulated protein-78; ATF6, activating transcription factor 6; CONTR, control; LPS, lipopolysaccharide; Trp, tryptophan. The bands represented Western blot images of GRP78 and ATF6. The data are presented as means ± SEM. CONTR vs. LPS was employed to obtain the response to LPS challenge. Linear and quadratic polynomial contrasts were employed to obtain the response of tryptophan supplementation in LPS-challenged piglets. Note: L (linear), Q (quadratic).

**Figure 3:**
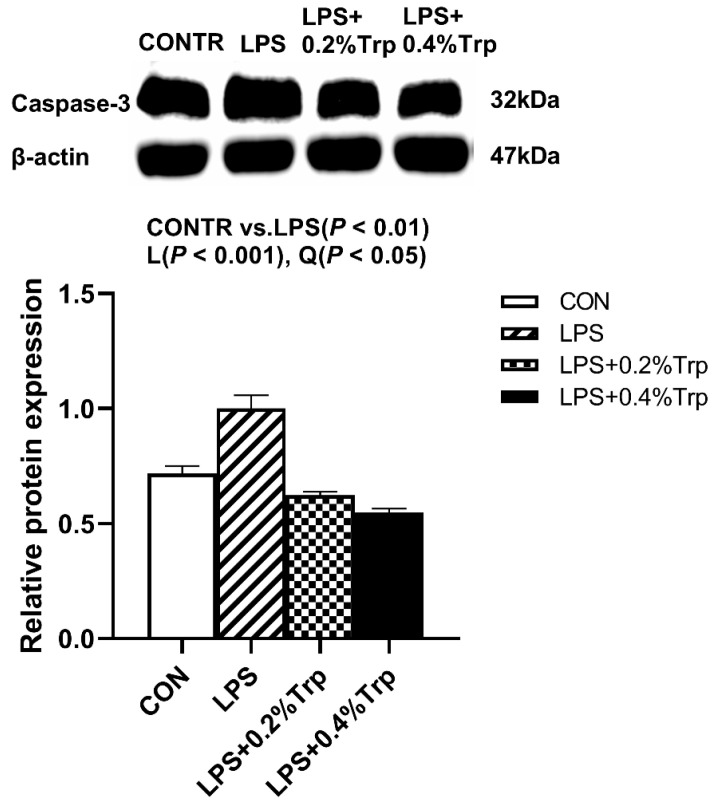
Impact of tryptophan supplementation on the ratio of caspase-3/β-actin after 4 h of *E. coli* LPS challenge in ileal mucosa of weaning piglets. CONTR, control; LPS, lipopolysaccharide; Trp, tryptophan. The band shown is the representative Western blot images of caspase-3. The data are presented as means ± SEM. CONTR vs. LPS was employed to obtain the response to LPS challenge. Linear and quadratic polynomial contrasts were employed to obtain the response of tryptophan supplementation in LPS-challenged piglets. Note: L (linear), Q (quadratic).

**Figure 4:**
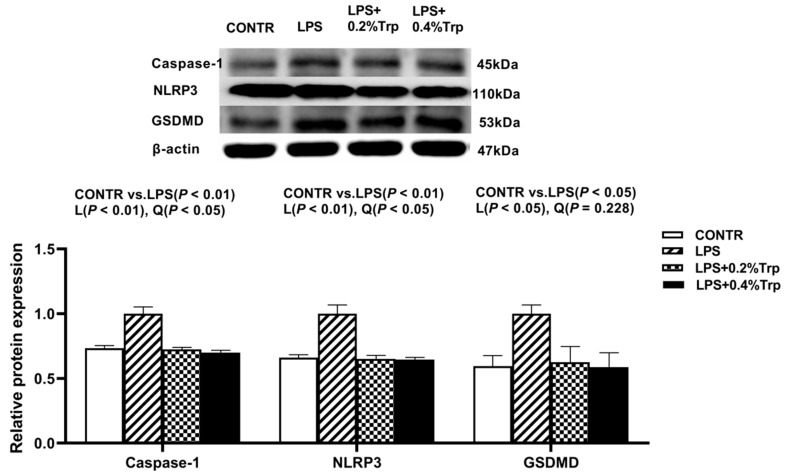
Impact of tryptophan supplementation on the ratios of caspase-1/β-actin, NLRP3/β-actin, and GSDMD/β-actin after 4 h of *E. coli* LPS challenge in ileal mucosa of weaning piglets. NLRP3, nucleotide-binding oligomerization domain-like receptor protein 3; GSDMD, gasdermin-D CONTR, control; LPS, lipopolysaccharide; Trp, tryptophan. The bands represented Western blot images of caspase-1, NLRP3, and GSDMD. The data are presented as means ± SEM. CONTR vs. LPS was employed to obtain the response to LPS challenge. Linear and quadratic polynomial contrasts were employed to obtain the response of tryptophan supplementation in LPS-challenged piglets. Note: L (linear), Q (quadratic).

Impact of tryptophan supplementation on expressions of ER stress-, apoptosis-, and pyroptosis-related protein in ileal tissues are shown in Figure 2. The LPS pigs had higher ratios of GRP78/β-actin, ATF6/β-actin (*p* < 0.01) (Figure 2), caspase-3/β-actin (*p* < 0.01) (Figure 3), caspase-1/-βactin (*p* < 0.01), NLRP3/β-actin (*p* < 0.01) and GSDMD/β-actin (*p* < 0.05) (Figure 4) in ileum than CONTR pigs. Relative to LPS pigs, the LPS + 0.2% Trp and LPS + 0.4% Trp pigs had lower ratios of GRP78/β-actin (linear, *p* < 0.001; quadratic, *p* < 0.01), ATF6/β-actin (linear, *p* < 0.001) (Figure 2), caspase-3/β-actin (linear, *p* < 0.001; quadratic, *p* < 0.05) (Figure 3), caspase-1/β-actin (linear, *p* < 0.01; quadratic, *p* < 0.05), NLRP3/β-actin (linear, *p* < 0.01; quadratic, *p* < 0.05), and GSDMD/β-actin (linear, *p* < 0.05) (Figure 4) in the ileum.

## 4. Discussion

In this study, a 0.2% dose of tryptophan supplementation significantly increased average daily gain and average daily feed intake. A 0.4% dose of tryptophan supplementation significantly enhanced the average daily feed intake of weaned piglets. A total of 0.2% of tryptophan groups had better growth performance than 0.4% of tryptophan before LPS challenge (data not shown). This is in agreement with the result of a previous study [12]. Stress can cause intestinal barrier dysfunction. Tryptophan can decrease stress and improve intestinal health, but the precise mechanism has been fully unclear. Tryptophan can enhance intestinal morphology, indicated by greater villus height, villus area, and smaller crypt depth. This result was in agreement with that of a previous study [22]. Tryptophan supplementation can enhance the antioxidant status and decrease the mRNA expression and concentration of proinflammatory cytokines after LPS challenge. These results were consistent with those of previous studies [9,10,12]. Inflammation can cause the catabolism of tryptophan in piglets. Tryptophan catabolism is mediated by tryptophan dioxygenase activation mainly in the liver or by IDO inducible by bacterial products (such as lipopolysaccharide) and proinflammatory mediators (such as TNF-α and IFN-γ) in several tissues [23]. Increased catabolism of tryptophan through the IDO may limit the use of tryptophan for growth-related protein synthesis [24]. In this experiment, LPS increased the protein content of IDO and IFN-γ in the ileum, suggesting that the catabolism of tryptophan could be stimulated under stress. In addition, we also found that the protein content of IDO and IFN-γ in the ileum of piglets supplemented with tryptophan were decreased after LPS challenge. This result indicated that tryptophan supplementation may alleviate the catabolism of tryptophan. In a related study, Le Floc’h et al. found that inflammation increases tryptophan catabolism and reduces the availability of tryptophan for growth [25]. Taken together, these results suggested that dietary tryptophan supplementation can contribute to gut health.

To extend the understanding of the beneficial effect of tryptophan in alleviating intestinal disorders, we studied its role in the regulation of ER stress, apoptosis, and pyroptosis signaling pathway. Ero1 has two isoforms, namely, Ero1*α* and Ero1*β*, which are important causes of intracellular ROS production [26]. Excessive ROS production will disturb the intracellular redox state and cause ER stress, which can destroy intestinal homeostasis and cause the progression of digestive disfunction in the host [27]. This notion has been verified by our results, in which LPS can increase Ero1*α*, Ero1*β*, and inflammatory response, reduce antioxidant status, and cause ER stress. ER stress is sensed by three unfolded protein response signal transducers, such as inositol-requiring enzyme 1 alpha (IRE1), protein kinase RNA-like endoplasmic reticulum kinase, and activating transcription factor 6 (ATF6) [28]. All three sensors were inhibited by attaching to the ER chaperone of GRP78 under non-stressed conditions. Under ER stress, GRP78 separated from the intraluminal domains of IRE1, protein kinase RNA-like endoplasmic reticulum kinase, or ATF6, and activated downstream cascade signaling [29]. CHOP can act as a junction for the entire ER stress sensor [30]. In the current study, LPS increased the expression of GRP78, ATF6, and CHOP. However, tryptophan can reverse the above-mentioned results induced by LPS. Thus, tryptophan supplementation can decrease ER stress. Currently, the effect of tryptophan supplementation on ER stress in pigs has not been studied. When ER stress occurs, CHOP is induced to be expressed in a large amount to induce apoptosis [31]. In the present study, tryptophan decreased the pro-apoptotic Bax expression and enhanced the anti-apoptotic Bcl-2 expression. Tryptophan supplementation also reduced the expression of caspase-3. Therefore, tryptophan reduced cell apoptosis.

ROS can also cause cell pyroptosis, which is a newly regarded form of inflammatory cell death. Pathogen-associated molecular patterns (PAMPs) or damage-associated molecular patterns (DAMPs) signals were identified by NLRP3. NLRP3 binds to the adaptor protein apoptosis-related speckle-like protein (ASC) and caspase-1. Then, the effect protein GSDMD is cleaved by activated caspase 1, developing cell membrane pores and releasing out the cells of the intracellular contents of proinflammatory cytokines IL-1β and IL-18 through the pores caused by the N-terminal domain of GSDMD [32,33]. Pyroptosis is casually linked to various diseases, such as intestinal dysfunction [34,35]. In the present study, we investigated that tryptophan reversed the expression of NLRP3, ASC, caspase 1, GSDMD, IL-1β, and IL-18 after LPS challenge, suggesting the beneficial effects of tryptophan supplementation in inhibiting pyroptosis signaling pathway. Until now, the effect of tryptophan supplementation on pyroptosis has not been studied.

## 5. Conclusions

The results suggest that tryptophan supplementation can enhance intestinal health as indicated by enhancing the antioxidant status and inhibiting inflammation, ER stress, apoptosis, and pyroptosis. Our study not only offers new information on the nutritional role of tryptophan but also deeply reveals the mechanism of tryptophan in regulating gut health in LPS challenges.

## Figures and Tables

**Table 1:** Ingredient composition of experimental diets (as-fed basis).

Ingredient	Content (%)	
	7–11 kg	11–25 kg
Corn	27.37	30.6
Extruded corn	30.84	32
Soybean oil	2.5	1.4
glucose	2	2
Whey powder	5	5
Dehulled soybean meal	13.24	13.04
Soybean protein concentrate	5	5
Extruded soybean	7	5
Fish meal	3	2.5
l-Lysine-HCl	0.52	0.44
dl-Methionine	0.11	0.08
l-Threonine	0.2	0.15
l-Tryptophan	0.03	0.01
l-Alanine	0.46	0.32
Choline chloride	0.15	0.15
Limestone	0.68	0.41
Monocalcium phosphate	1.35	1.35
NaCl	0.25	0.25
Vitamin premix ^a^	0.05	0.05
Mineral premix ^b^	0.25	0.25
Total	100	100
**Nutrient level ^c^**		
Digestible energy	3.55 Mcal/kg	3.49 Mcal/kg
Crude protein	19.72%	18.65%
Calcium	0.80%	0.68%
Total phosphorus	0.66%	0.64%
Available phosphorus	0.48%	0.46%
SID-Lysine	1.36%	1.24%
SID-Methionine	0.40%	0.36%
SID-Threonine	0.80%	0.73%
SID-Tryptophan	0.23%	0.20%

^a^ The vitamin premix provides the following per kilogram of diet: VA 15000 IU; VD_3_ 5000 IU; VE 40 IU; VK_3_ 5 mg; VB_1_ 5 mg; VB_2_ 12.5 mg; VB_6_ 6 mg; VB_12_ 600 μg; d-pantothenic acid 25 mg; nicotinic acid 50 mg; folic acid 2.5 mg; biotin 2.5 mg. ^b^ The mineral premix provides the following per kilogram of diet: copper (CuSO_4_·H_2_O) 6 mg; iron (FeSO_4_·H_2_O) 100 mg; zinc (ZnSO_4_·H_2_O) 100 mg; manganese (MnSO_4_·H_2_O) 4 mg; iodine (KI) 0.14 mg; selenium (Na_2_SeO_3_) 0.3 mg. ^c^ Nutrient levels are calculated values.

**Table 2:** Primer sequences used for real-time PCR.

Target Genes	Forward Primer	Reverse Primer	Accession Number	Temperature (°C)	Product Size (bp)
TNF-α	CGACTCAGTGCCGAGATCAA	GACCTGCCCAGATTCAGCAA	JF831365.1	58	60
IL-1β	AAGGCCGCCAAGATATAACTGA	GCCCTCTGGGTATGGCTTTC	NM_001302388.1	58	71
IL-6	ATGCTTCCAATCTGGGTTCAA	CACAAGACCGGTGGTGATTCT	AF518322.1	58	61
IL-8	ACATCCATGAGGAAGACAGTTTGA	CGGGAACTCCACGCTAGATTC	AB057440.1	58	70
IL-18	AGGGACATCAAGCCGTGTTT	CGGTCTGAGGTGCATTATCTGA	AY450287.1	58	66
GRP78	GAGATCATCGCCAACGATCA	CAGGAGTGAAGGCCACATATGAC	XM_001927795.5	58	61
CHOP	CTCCAGATTCCAGTCAGAGTTCTAT	TCTCCTGCTCCTTCTCCTTCAT	NM_001144845.1	58	60
ATF6	TCAGGGACCTGCCAAGTGA	GGGTCAATGAGTGAAGAGAAAGC	XM_021089515.1	58	68
ERO1α	CGGCGCAGAGGTGCTT	CAACATCACAGGTACAATCATCCA	NM_001137627.1	58	60
ERO1β	GGGCAAGATACGATGATTCACA	TACTGAGCAGCTGGCGATCTC	XM_013981626.2	58	50
ASC	CCAAGCCAGCTGGAATCAA	TGCAGTGCTGGTTTGTTGTCT	MK302492.1	58	58
GSDMD	GGCAGCGTCATTGCATTTC	TGAAGGTTCGCTGCTTCTTGT	XM_021090504.1	58	91
NLRP3	TCCCCTGGTCTGCTGGATT	ACTCTTGCCGCTATCCATCTG	NM_001256770.2	58	61
Caspase-1	CGACCCCCACCTTGCA	AAGGCATTTTCCAGATTGTGAAC	NM_214162.1	58	61
Caspase-3	CCGGAATGGCATGTCGAT	TGAAGGTCTCCCTGAGATTTGC	NM_214131.1	58	60
Bax	AAGCGCATTGGAGATGAACTG	CACGGCTGCGATCATCCT	XM_003127290.5	58	59
BCL2	CCAGCATGCGGCCTCTAT	GACTGAGCAGCGCCTTCAG	AB271960.1	58	57
β-actin	TGCGGGACATCAAGGAGAA	GCCATCTCCTGCTCGAAGTC	DQ452569.1	58	59

TNF-α, tumor necrosis factor-α; IL-1β, interleukin-1β; IL-6, interleukin-6; IL-8, interleukin-8; IL-18, interleukin-18; ERO1α, endoplasmic reticulum oxidoreductase-1α; GRP78, glucose-regulated protein-78; ATF6, activating transcription factor 6; ERO1β, endoplasmic reticulum oxidoreductase-1β; NLRP3, nucleotide-binding oligomerization domain-like receptor protein 3; GSDMD, gasdermin-D; ASC, apoptosis-associated speck-like protein containing a CARD; BCL2, B-cell lymphoma-2; Bax, BCL2-associated X protein; CHOP, C/EBP homologous protein.

**Table 3:** Effect of tryptophan supplementation on ileal morphology after 4 h LPS challenge in piglets.

Item	Treatment ^1^				SEM	*p-*Value ^2^		
	CONTR	LPS	LPS + 0.2% Trp	LPS + 0.4% Trp		CONTR vs. LPS	Linear	Quadratic
Villus height, μm	401.51	193.11	320.62	341.93	19.83	<0.001	0.001	0.108
Villus area, μm^2^	237,721.19	101,927.17	177,673.28	196,176.90	14,306.24	0.001	0.004	0.258
Crypt depth, μm	254.97	274.17	212.78	191.96	11.60	0.606	0.002	0.297
VCR	1.76	0.77	1.63	1.92	0.12	0.001	<0.001	0.165

SEM, standard error of mean. VCR, villus height/crypt depth ratio; LPS, lipopolysaccharide; Trp, tryptophan. ^1^ CONTR (non-challenged control), piglets were supplemented with a basal diet and injected with 0.9% NaCl solution; LPS (LPS-challenged control), piglets were supplemented with the same basal diet and injected with *E. coli* LPS; LPS + 0.2% Trp, piglets fed a 0.2% tryptophan-supplemented diet as well as injected with LPS; LPS + 0.4% Trp, piglets fed a 0.4% tryptophan-supplemented diet as well as injected with LPS. ^2^ CONTR vs. LPS was employed to obtain the response of LPS challenge. Linear and quadratic polynomial contrasts were employed to obtain the response of tryptophan supplementation in LPS-challenged piglets.

**Table 4:** Impact of tryptophan on ileal antioxidant capacity after 4 h LPS challenge in piglets.

Item	Treatment ^1^				SEM	*p-*Value ^2^		
	CONTR	LPS	LPS + 0.2% Trp	LPS + 0.4% Trp		CONTR vs. LPS	Linear	Quadratic
MDA, nmol/mg prot	0.49	1.01	0.62	0.81	0.05	<0.001	0.069	0.004
T-SOD, U/mg prot	75.51	51.21	67.34	64.37	2.72	0.015	0.008	0.022
CAT, U/mg prot	9.24	6.02	8.45	8.64	0.34	0.007	<0.001	0.015
GSH-Px, U/mg prot	16.48	2.87	16.30	9.93	1.21	<0.001	<0.001	<0.001
H_2_O_2_, U/mg prot	0.48	0.88	0.47	0.59	0.04	<0.001	<0.001	<0.001
ROS, pg/mg prot	0.49	1.01	0.62	0.81	1.97	<0.001	<0.001	<0.001

SEM, standard error of mean. MDA, malondialdehyde; CAT, catalase; GSH-Px, glutathione peroxidase; T-SOD, total superoxide dismutase; H_2_O_2_, hydrogen peroxide; ROS, reactive oxygen species; LPS, lipopolysaccharide; Trp, tryptophan. ^1^ CONTR (non-challenged control), piglets were supplemented with a basal diet and injected with 0.9% NaCl solution; LPS (LPS-challenged control), piglets were supplemented with the same basal diet and injected with *E. coli* LPS; LPS + 0.2% Trp, piglets fed a 0.2% tryptophan-supplemented diet as well as injected with LPS; LPS + 0.4% Trp, piglets fed a 0.4% tryptophan-supplemented diet as well as injected with LPS. ^2^ CONTR vs. LPS was employed to obtain the response of LPS challenge. Linear and quadratic polynomial contrasts were employed to obtain the response of tryptophan supplementation in LPS-challenged pigs.

**Table 5:** Effects of tryptophan on the ileal cytokine concentration and activity of IDO after 4 h LPS challenge in piglets.

Item	Treatment ^1^				SEM	*p-*Value ^2^		
	CONTR	LPS	LPS + 0.2% Trp	LPS + 0.4% Trp		CONTR vs. LPS	Linear	Quadratic
TNF-α, pg/mg prot	33.15	64.52	35.19	41.92	2.99	<0.001	<0.001	<0.001
IL-1β, pg/mg prot	123.98	179.53	93.65	110.62	7.43	<0.001	<0.001	<0.001
IL-6, pg/mg prot	172.47	257.39	135.52	171.16	10.33	<0.001	<0.001	<0.001
IL-8, pg/mg prot	12.25	18.43	8.79	10.17	0.86	<0.001	<0.001	<0.001
IL-10, pg/mg prot	76.14	49.78	67.38	95.13	3.91	<0.001	<0.001	0.150
IDO, pg/mg prot	9.20	12.11	6.72	10.39	0.48	<0.001	0.008	<0.001
IFN-γ, pg/mg prot	14.76	21.07	11.36	18.39	0.89	<0.001	0.019	<0.001

SEM, standard error of mean; TNF-α, tumor necrosis factor-α; IL-1β, interleukin-1β; IL-6, interleukin-6; IL-8, interleukin-8; IL-10, interleukin-10; IDO, indoleamine 2, 3 dioxygenase; IFN-γ, interferon-γ; LPS, lipopolysaccharide; Trp, tryptophan. ^1^ CONTR (non-challenged control), piglets fed a basal diet and injected with 0.9% NaCl solution; LPS (LPS-challenged control), piglets fed the same basal diet and injected with *E. coli* LPS; LPS + 0.2% Trp, piglets fed a 0.2% tryptophan-supplemented diet as well as injected with LPS; LPS + 0.4% Trp, piglets fed a 0.4% tryptophan-supplemented diet as well as injected with LPS. ^2^ CONTR vs. LPS was employed to obtain the response of LPS challenge. Linear and quadratic polynomial contrasts were employed to obtain the response of tryptophan supplementation in LPS-challenged pigs.

**Table 6:** Impact of tryptophan on the ileal proinflammatory cytokines-related gene expressions after 4 h LPS challenge in piglets.

Item	Treatment ^1^				SEM	*p-*Value ^2^		
	LPS	CONTR	LPS + 0.2% Trp	LPS + 0.4% Trp		CONTR vs. LPS	Linear	Quadratic
TNF-α	1.00	0.17	0.16	0.13	0.08	<0.001	<0.001	<0.001
IL-1β	1.00	0.55	0.51	0.59	0.05	<0.001	0.002	0.007
IL-6	1.00	0.75	0.76	0.79	0.03	0.001	0.002	0.01
IL-8	1.00	0.65	0.29	0.31	0.06	<0.001	<0.001	<0.001
IL-18	1.00	0.34	0.35	0.37	0.06	<0.001	<0.001	<0.001

SEM, standard error of mean; TNF-α, tumor necrosis factor-α; IL-1β, interleukin-1β; IL-6, interleukin-6; IL-8, interleukin-8; LPS, lipopolysaccharide; Trp, tryptophan. ^1^ CONTR (non-challenged control), piglets were supplemented with a basal diet and injected with 0.9% NaCl solution; LPS (LPS-challenged control), piglets were supplemented with the same basal diet and injected with *E. coli* LPS; LPS + 0.2% Trp, piglets fed a 0.2% tryptophan-supplemented diet as well as injected with LPS; LPS + 0.4% Trp, piglets fed a 0.4% tryptophan-supplemented diet as well as injected with LPS. ^2^ CONTR vs. LPS was employed to obtain the response of LPS challenge. Linear and quadratic polynomial contrasts were employed to obtain the response of tryptophan supplementation in LPS-challenged piglets.

**Table 7:** Impact of tryptophan on the ileal endoplasmic reticulum stress, pyroptosis-, and apoptosis-related gene expressions after 4 h LPS challenge in piglets.

Item	Treatment ^1^				SEM	*p-*Value ^2^		
	LPS	CONTR	LPS + 0.2% Trp	LPS + 0.4% Trp		CONTR vs. LPS	Linear	Quadratic
GRP78	1.00	0.61	0.72	0.79	0.03	<0.001	0.001	0.001
CHOP	1.00	0.62	0.40	0.41	0.05	<0.001	<0.001	<0.001
ATF6	1.00	0.18	0.23	0.31	0.07	<0.001	<0.001	<0.001
ERO1α	1.00	0.52	0.57	0.72	0.04	0.001	0.002	<0.001
ERO1β	1.00	0.66	0.76	0.71	0.03	<0.001	<0.001	0.049
Caspase-3	1.00	0.45	0.48	0.48	0.05	<0.001	<0.001	<0.001
Bax	1.00	0.46	0.44	0.47	0.05	<0.001	<0.001	<0.001
BCL2	1.00	1.49	1.62	1.56	0.07	0.012	0.001	0.012
ASC	1.00	0.06	0.08	0.1	0.08	<0.001	<0.001	<0.001
GSDMD	1.00	0.17	0.16	0.13	0.38	<0.001	<0.001	<0.001
NLRP3	1.00	0.64	0.68	0.57	0.04	<0.001	<0.001	0.047
Caspase-1	1.00	0.16	0.19	0.25	0.36	<0.001	<0.001	<0.001

SEM, standard error of mean; ERO1*α*, endoplasmic reticulum oxidoreductase-1α; GRP78, glucose-regulated protein-78; ATF6, activating transcription factor 6; ERO1*β*, endoplasmic reticulum oxidoreductase-1β; NLRP3, nucleotide-binding oligomerization domain-like receptor protein 3; GSDMD, gasdermin-D; ASC, apoptosis-associated speck-like protein containing a CARD; BCL2, B-cell lymphoma-2; Bax, BCL2-associated X protein; CHOP, C/EBP homologous protein; LPS, lipopolysaccharide; Trp, tryptophan. ^1^ CONTR (non-challenged control), piglets were supplemented with a basal diet and injected with 0.9% NaCl solution; LPS (LPS-challenged control), piglets were supplemented with the same basal diet and injected with *E. coli* LPS; LPS + 0.2% Trp, piglets fed a 0.2% tryptophan-supplemented diet as well as injected with LPS; LPS + 0.4% Trp, piglets fed a 0.4% tryptophan-supplemented diet as well as injected with LPS. ^2^ CONTR vs. LPS was employed to obtain the response of LPS challenge. Linear and quadratic polynomial contrasts were employed to obtain the response of tryptophan supplementation in LPS-challenged piglets.

## Data Availability

Data is contained within the article.
